# Artificial Intelligence (AI) Applications for Point of Care Ultrasound (POCUS) in Low-Resource Settings: A Scoping Review

**DOI:** 10.3390/diagnostics14151669

**Published:** 2024-08-01

**Authors:** Seungjun Kim, Chanel Fischetti, Megan Guy, Edmund Hsu, John Fox, Sean D. Young

**Affiliations:** 1 Department of Informatics, University of California, Irvine, CA 92697, USA; syoung5@hs.uci.edu; 2 Department of Emergency Medicine, Brigham and Women’s Hospital, Boston, MA 02115, USA; 3 Department of Emergency Medicine, University of California, Irvine, CA 92697, USA; guym@hs.uci.edu (M.G.); edhsu@hs.uci.edu (E.H.); jfox@hs.uci.edu (J.F.)

**Keywords:** point-of-care ultrasound (POCUS), artificial intelligence (AI), low-resource settings, resource-limited settings, low- or middle-income countries, rural, remote

## Abstract

Advancements in artificial intelligence (AI) for point-of-care ultrasound (POCUS) have ushered in new possibilities for medical diagnostics in low-resource settings. This review explores the current landscape of AI applications in POCUS across these environments, analyzing studies sourced from three databases—SCOPUS, PUBMED, and Google Scholars. Initially, 1196 records were identified, of which 1167 articles were excluded after a two-stage screening, leaving 29 unique studies for review. The majority of studies focused on deep learning algorithms to facilitate POCUS operations and interpretation in resource-constrained settings. Various types of low-resource settings were targeted, with a significant emphasis on low- and middle-income countries (LMICs), rural/remote areas, and emergency contexts. Notable limitations identified include challenges in generalizability, dataset availability, regional disparities in research, patient compliance, and ethical considerations. Additionally, the lack of standardization in POCUS devices, protocols, and algorithms emerged as a significant barrier to AI implementation. The diversity of POCUS AI applications in different domains (e.g., lung, hip, heart, etc.) illustrates the challenges of having to tailor to the specific needs of each application. By separating out the analysis by application area, researchers will better understand the distinct impacts and limitations of AI, aligning research and development efforts with the unique characteristics of each clinical condition. Despite these challenges, POCUS AI systems show promise in bridging gaps in healthcare delivery by aiding clinicians in low-resource settings. Future research endeavors should prioritize addressing the gaps identified in this review to enhance the feasibility and effectiveness of POCUS AI applications to improve healthcare outcomes in resource-constrained environments.

## 1. Introduction

The global diagnostic ultrasound market has seen steady growth, reaching a value of USD 7.39 billion in 2023, with projections expecting it to reach approximately USD 11 billion by 2033 [1,2]. This growth stems from the strengths of ultrasonography being portable, affordable, and radiation-free, unlike computed tomography (CT) [3]. Point-of-care-ultrasound (POCUS) refers to ultrasound performed by the clinician at the bedside of their patient. Despite concerns that its portability might compromise performance, POCUS machines largely retain conventional ultrasound features and perform comparably well [4,5]. POCUS holds immense potential to make medical care more accessible, even in the most austere conditions, owing to its small size, portability, and affordability. This makes it an invaluable tool in places with limited resources. Accordingly, the use of POCUS has been widely adopted in various resource-limited settings, such as developing countries and conflict zones, areas affected by war or political instability that disrupt essential services such as housing, transportation, communication, sanitation, water, and healthcare [6,7,8]. In this review, low-resource setting refers to, but is not limited to, environments in which resources for high-quality healthcare (e.g., finances, trained personnel, medical equipment, computing resources) are constrained [9,10]. Specifically, this review focuses on the following low-resource areas: rural or remote [11], low- and middle-income countries (LMICs) [7,12], emergency contexts [13], and environments lacking key resources [14].

Artificial intelligence (AI) optimizes processes through automation and in-depth analyses surpassing human capability and, thus, has important implications for POCUS used in low-resource settings. As ultrasound machines become more ubiquitous and portable, more clinicians will continue to adopt ultrasound as the preferred diagnostic and/or therapeutic modality. This, however, leaves a potential area and gap in medical training and education. It is within this space that AI presents a unique opportunity to facilitate both image acquisition and image interpretation when technology outstrips human skill levels.

Because technology has evolved so rapidly within the last decade, there have been limited studies on the applications and developments of AI for POCUS, specifically for POCUS used in or developed for low-resource settings. Previous literature mainly focuses on POCUS education and training, aiming to nurture proficient POCUS practitioners or enhance the acceptance and utilization of POCUS in such settings [15,16,17]. Advanced technologies including telehealth applications using POCUS for both diagnosis and remote education have also been proposed but were irrelevant to AI [18,19,20]. Some articles related to AI were either on conventional ultrasound but not POCUS or pertinent to broad and general situations but not particularly to low-resource settings [21,22,23,24,25]. This review aims to accomplish two research objectives: (1) to examine the current state of POCUS AI applications in and for low-resource settings using various levels of analysis, including target population, geography or country, type of low-resource setting, and the objective and implication of the study; and (2) to identify limitations and barriers that those AI systems face to leave them for future studies to address.

## 2. Materials and Methods

This paper utilized the Cochrane guidelines for conduct and the Preferred Reporting Items for Systematic Reviews and Meta-Analyses for Scoping Reviews (PRISMA-ScR) guidelines to minimize bias and provide the review with more structure. The approval of the Institutional Review Board (IRB) was not necessary as the study did not involve human participants.

A comprehensive search was conducted on three electronic databases (SCOPUS, PubMed, and Google Scholars) in June 2024 using the following keywords: ((POCUS) OR (Point-of-care ultrasound) OR (Portable Ultrasound)) AND ((AI) OR (Artificial Intelligence) OR (Machine Learning) OR (Deep Learning) OR (NLP) OR (Natural Language Processing) OR (Large Language Model) OR (LLM) OR (Generative AI)) AND ((low-resource) OR (resource-limited) OR (rural) OR (remote) OR (austere setting) OR (LMIC) OR (Low-middle income countries) OR (military) OR (space) OR ((emergency) AND (low-resource))). A data-charting form was jointly developed by all authors to determine which variables to extract. The actual extraction of metadata was conducted by two authors (SK and SY). Such metadata included authors, population, geography or country, type of low-resource settings, type of AI, and research objectives. We did not impose a time restriction to ensure the search was systematic [26,27]. The records retrieved from these databases were exported to Covidence (Melbourne, Australia) a platform that aids scholars with literature reviews [28]. After duplicates were eliminated, the records went through two stages of screening.

**Figure 1:**
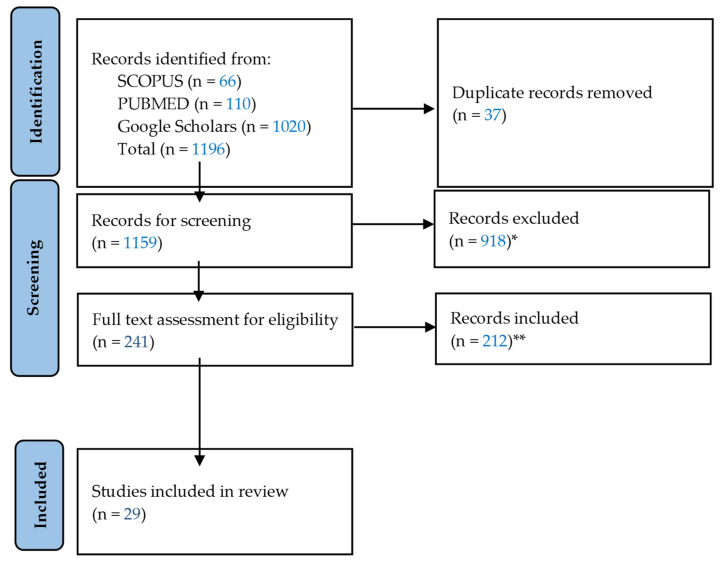
**PRISMA flow diagram.** * not peer-reviewed or non-journals (*n* = 143); reviews (*n* = 197); not POCUS-related or irrelevant to ultrasound (*n* = 214); not low-resource setting (*n* = 256); not AI-related (*n* = 108). ** not POCUS-related or irrelevant to ultrasound (*n* = 52); not low-resource setting (*n* = 143); not AI-related (*n* = 17).

During the first stage, the title, abstract, and type of study were examined and a total of 548 were excluded. A more specific breakdown is available in Figure 1. This stage was intended to filter out the articles meeting the exclusion criteria and deemed ineligible based on the title and abstract. More specifically, articles covering non-ultrasound applications, topics irrelevant to low-resource settings and AI, manuscripts that were not peer-reviewed, non-journal pieces (e.g., books), non-English articles, reviews, and any documents generated by non-humans (e.g., ChatGPT) were not included.

During the second stage, records went through a full-text review. The same exclusion criteria used in the first stage of screening were equally applied but this time on a full-text basis. In addition to the studies that used or tested AI applications in low-resource settings, manuscripts that explicitly alluded to the potential benefits and usefulness of the proposed AI applications in low-resource settings were also included in our scoping review. Both stages of screening were conducted based on the inclusion and exclusion criteria in Table 1. All authors were involved in both stages of the screening process. Conflict resolution when disagreements arose was conducted jointly by all six authors. Determination of whether each of the articles extracted was relevant and maintained high enough quality was based on sufficient discussions among all authors. The protocol used in this review was not preregistered.

## 3. Results

### 3.1. Results

A total of 1196 records were retrieved, 37 duplicates were removed, and 918 records were removed after the initial screening. The remaining 241 records underwent full-text reviews according to the inclusion and exclusion criteria detailed in Table 1, resulting in 29 unique studies. Figure 1 displays the PRISMA flow diagram, which visualizes this screening process. Table 2 showcases the metadata of the 29 studies included in this review. The majority of studies (79%) were conducted from 2021 to 2023. The most frequently addressed medical departments were pulmonology (31%), obstetrics (21%), emergency medicine or intensive care units (ICU) (14%), and cardiology (14%). Deep learning was the most commonly used AI technique, employed in 23 studies (79%) to enhance the operation of POCUS in resource-limited settings. Other AI techniques utilized were machine learning, computer vision, and Bayesian machine learning.

### 3.2. Types of Populations and Locations

Almost half of the studies (45%) did not specify target populations. The population column in Table 2 is labeled “N/A” for these studies. Instead of focusing on particular populations, these studies proposed and assessed high-level AI algorithms or architecture that can automatically measure medical entities (e.g., bladder volume), assist in diagnosing or classifying conditions, and improve quality assurance of operations related to POCUS in low-resource environments. Examples of such health measurements included left ventricular ejection fractions and bladder volume [31,46]. Conditions for automatic POCUS image-based diagnosis varied from pneumothorax to COVID-19 and breast cancer [33,37,44,49]. Other populations covered in the remaining studies included infants or neonates (14%), pregnant women (17%), and COVID-19 patients (17%). In regards to the location of research, the United States was where most of research studies (45%) were conducted followed by Canada (21%). Other countries included Vietnam, India, Zambia, South Korea, Egypt, Norway, and Ethiopia.

### 3.3. Types of Low-Resource Settings

Four broad categories were identified regarding the types of low-resource settings: LMIC, rural or remote, emergency, and lack of key resources. Seven studies (24%) pertained to rural and remote settings. Two studies (7%) focused on emergency situations. Ten studies (34%) targeted LMICs.

A total of 18 studies (62%) aimed to address limitations due to the scarcity of key resources. Key resources included experienced personnel, computing resources, and data for training AI models. Cho et al. developed a deep learning-based system to measure bladder volume from POCUS images. This system was designed to operate on devices with limited computing power, which is typical in LMICs and rural areas [31]. This may aid clinicians in assessing bladder volume even in low-resource settings with limited access to complex equipment.

Baloescu et al. addressed the lack of experienced staff with sonography experience needed to assess B-lines in point-of-care lung ultrasound, which is crucial for diagnosing shortness of breath in the emergency department (ED) [44]. The study developed and evaluated a deep convolutional neural network-based deep learning algorithm that quantified the assessment of B-lines in lung ultrasound by utilizing 400 ultrasound clips from an existing database of ED patients. The model achieved a decent performance of 93% sensitivity and 96% specificity in identifying B-lines compared with expert evaluations, suggesting that the system could empower inexperienced personnel in low-resource hospitals to perform B-line identification and quantification, which may be challenging for novice users.

To address the lack of data for training AI systems for POCUS-related tasks, Blaivas et al. presented a new method of using unrelated ultrasound window data (only apical 4-chamber views) to train a POCUS machine learning algorithm to measure the left ventricular ejection fraction. This approach is expected to guide the development of future POCUS and deep learning algorithms to mitigate the data paucity common in LMICs.

## 4. Discussion

This review aims to understand the current landscape of AI applications for POCUS in low-resource settings. It seeks to identify gaps in these AI applications in order to inform future research and, ultimately, benefit both the clinicians and the patients in resource-constrained environments.

A major gap identified in the studies included in this review was the potential inability of AI systems to generalize to other health conditions, populations, or settings. With ongoing training and adjustments, the generalizability of ultrasound AI models is expected to improve. Many of the articles reviewed were based on pilot studies. Consequently, the experiments, conducted under restricted conditions, may not fully account for all variables in real-world scenarios. Nhat et al. presented an AI-enabled point-of-care lung ultrasound (LUS) solution that assists non-expert clinicians in LMIC intensive care units (ICU) with LUS interpretation [29]. The AI system, however, was only trained on data from patients with severe dengue or sepsis. Future studies, therefore, are needed to investigate whether this AI solution is equally helpful in interpreting point-of-care LUS images for other diseases. Libon et al. sought to assess the feasibility of implementing a US FDA-cleared AI screening device for developmental dysplasia of the hip (DDH) for infants ages 6 to 10 weeks [30]. This pilot study was limited in scale, involving 306 infants from a suburban Western Canadian area with a substantial Indigenous population. Researchers may want to initiate a separate study in the future that employs a greater number of infants with more racial and geographical diversity.

Furthermore, the performance of some algorithms proposed in the studies may diminish with more complex datasets. For example, Aujila et al. developed a machine learning framework to automatically diagnose neonatal lung pathologies in low-resource and, particularly, remote settings [34]. Linear discriminant analysis (LDA) was used as the main classifier algorithm, but for larger datasets, this linear classifier may not be the most appropriate. Therefore, deep learning-based classifiers that can capture more convoluted patterns may prove beneficial. Nevertheless, this simple linear classifier was selected over the more complex classifiers in this study to extract and interpret meaningful features relevant to clinical markers and keep the outcomes conservative and realistic. The trade-off between the interpretability and complexity of AI systems should be a key consideration for future research on this topic.

Regional disparities in research activities on the applications of AI for POCUS in low-resource settings may be concerning. Only 30% of the studies included in this review were conducted in LMICs. Even when some AI application is designed for low-resource settings, bringing it to resource-limited settings for testing and assessment is crucial for ensuring its usefulness in such settings. The concentration of studies in the U.S. and Canada suggests a need for increased research investment and collaboration in LMICs and other underserved regions to ensure that the benefits of AI applications for POCUS are globally accessible. Pokaprakarn et al. and Viswanathan et al. may serve as exemplary models to address this issue of regional disparities [52,54]. Researchers from both studies were based in the U.S. but proceeded with their testing and evaluation of the developed AI systems in not just the U.S. but also in Zambia.

Patient compliance and research ethics may be notably critical issues in studies conducted in remote settings. These challenges may arise because researchers and patients are not co-located, which complicates supervision, interaction, and rapport building. Sultan et al. performed a pilot analysis to evaluate the performance of AI-powered COVID-19 detection systems based on point-of-care lung ultrasound images [32]. This study primarily focused on inexperienced users, who comprise most of the workforce in low-resource settings. The study anticipates that patient compliance within the remotely monitored subgroup will be a significant limitation. Expected barriers to compliance include reluctance to self-administer daily POCUS due to discomfort, fear of inadequate care, and misunderstandings of the study protocols. Ensuring the security of ultrasound imaging data and other health records to protect patient privacy and confidentiality must be prioritized in future, larger-scale studies.

Future research must tackle the challenge of standardizing POCUS devices, protocols, and algorithms. Four popular handheld POCUS devices are currently available on the market: Butterfly iQ+ by Butterfly Network Inc. (Burlington, MA, USA), Kosmos by EchoNous (Redmond, WA, USA), Vscan Air by General Electric (Boston, MA, USA), and Lumify by Philips Healthcare (Andover, MA, USA). All of these devices have different functionalities and views with no single handheld ultrasound device perceived to have all the desired characteristics [58]. In one study evaluating the performance of deep learning algorithms on 21 videos obtained from each of the two novel POCUS machines, performance was significantly worse than the performance from a common POCUS machine in widespread use [59]. Lack of algorithm standardization also leads to degrading model performance. Blaivas et al. developed a “do-it-yourself” (DIY) deep learning algorithm for classifying POCUS images (pelvis, heart, lung, abdomen, musculoskeletal, ocular, and central vascular access) to enhance the quality assurance workflow for POCUS programs [43]. This algorithm, which processed ultrasound images from various POCUS programs, exhibited high-performance variability across different systems. This implied that the aforementoned algorithm would require further training on new image data samples when used in different POCUS programs. This algorithm has difficulty with classifying musculoskeletal ultrasound images, for instance, while performing well in other domains. Standardizing devices, protocols, and algorithms is crucial in resource-limited settings with limited options. A standardized all-in-one solution may be a better alternative.

The diversity of POCUS AI applications across different domains, including lung, hip, and bladder, illustrates the challenges of tailoring solutions to meet the specific needs of each application. For instance, the ability of AI to enhance diagnostic precision through the quantitative measurement of DDH in infants showcases the direct and reproducible benefits of AI in well-defined clinical measures in hip dysplasia screening, as demonstrated by Libon et al. [30]. Similarly, bladder volume estimation using AI in low-resource settings exemplifies the potential for AI to provide significant operational efficiencies in routine diagnostics [31]. Conversely, lung ultrasound applications, such as those explored by Nhat et al. LUS in intensive care, present greater challenges due to the qualitative nature of assessments and the subtlety of visual cues, which impact the reproducibility and consistency of AI predictions [29]. These examples underscore the necessity for AI systems that are specifically adapted to the complexities of each medical imaging domain, ensuring that AI tools augment clinical workflows effectively without leading to misinterpretation or overreliance. By analyzing the impact separately by application area, researchers will better understand the distinct impacts and limitations of AI, aligning research and development efforts with the unique characteristics of each clinical condition.

This review is not without limitations. The protocol was not preregistered as mentioned in the Methods section. Preregistration of the review protocol will be desirable for similar future studies to ensure further rigor and consistency in the protocol. Furthermore, readers of this review may encounter difficulties in applying the insights drawn from this review due to the broad scope of applications covered in this review. Future research may warrant focusing on applications for specific departments (e.g., cardiology) so that the role of AI systems for POCUS may be robustly validated, at least for that particular department or domain of application.

## 5. Conclusions

This review examined the current state of AI in POCUS, employing filters such as medical departments, countries, research geographies, AI types, and low-resource settings. The limitations of various POCUS AI applications, implemented and evaluated in low-resource settings, were extensively analyzed. Identified limitations include limited generalizability, insufficient datasets for training AI systems, regional disparities in research on AI applications for POCUS, potential patient noncompliance, ethical challenges in remote settings, and a lack of standardized POCUS protocols, algorithms, and devices. Despite these challenges, the findings demonstrate that POCUS AI systems are both feasible and effective in aiding patients and clinicians to overcome barriers such as scarce computing resources and a lack of trained personnel in low-resource settings. Future research should focus on developing new POCUS AI applications that both address the gaps identified in this review and prove cost-effective, using fewer computational resources without sacrificing performance. Lastly, if new POCUS AI applications could become more user-friendly, this would effectively empower the most inexperienced users in low-resource settings to perform point-of-care ultrasound with high fidelity.

## Figures and Tables

**Table 1:** Inclusion and exclusion criteria for screening.

Inclusion Criteria	Exclusion Criteria
- Point-of-care ultrasound used - Low-resource setting - artificial intelligence (AI) - application - Peer-reviewed - Not reviews	- Irrelevant to ultrasound or only related to traditional ultrasound - Not low-resource setting - Not English-speaking - Not human - Not artificial intelligence (AI)-related - Not peer-reviewed - Not journals (e.g., books) - Reviews (e.g., systematic reviews)

**Table 2:** Metadata of studies included in the review.

Author	Population	Geography/ Country	Low-Resource Setting Type/ Department	AI Used	Objective
Nhat et al., 2023 [29]	Doctors, clinicians	Vietnam	LMIC/Intensive care unit (ICU)	Deep learning	Develop an AI solution that assists lung ultrasound (LUS) practitioners, especially with LUS interpretation, and assess its usefulness in a low-resource ICU.
Libon et al., 2023 [30]	Infants	Canada	Remote/Pediatrics	US FDA-cleared artificial intelligence (AI) screening device for infant hip dysplasia (DDH)	Evaluate the feasibility of implementing an artificial intelligence-enhanced portable ultrasound tool for infant hip dysplasia (DDH) screening in primary care by determining its effectiveness in practice and evaluating patient and provider feedback.
Cho et al., 2023 [31]	N/A	South Korea	Lack of computing resources/Urology	Deep learning	Develop a system for measuring bladder volume in ultrasound images that could be used in point-of-care settings. Create a system based on deep learning optimized for low-resource system-on-chip (SoC) due to its speed and accuracy, even on devices with limited computing power. This could improve bladder disorder diagnosis by making bladder volume assessment easier in situations when access to complex equipment is limited.
Sultan et al., 2023 [32]	Clinicians, patients	United States	Remote/Pulmonology	Deep learning	Propose the use of teleguided POCUS supported by AI technologies for monitoring COVID-19 patients by non-experienced personnel, including self-monitoring by the patients themselves in a remote setting.
Perera et al., 2021 [33]	N/A	United States	Rural and LMIC/Pulmonology	Deep learning	Present an image-based solution that automatically tests for COVID-19. This will allow for rapid mass testing to be conducted with or without a trained medical professional, which can be applied to rural environments and third-world countries.
Aujla et al., 2023 [34]	N/A	Canada	Remote and LMIC/Pulmonology and neonatology	Machine learning	Propose an automated point-of-care tool for classifying and interpreting neonatal lung ultrasound (LUS) images, which will be useful in remote or developing countries with a lack of well-trained clinicians.
Abdel-Basset et al., 2022 [35]	N/A	Egypt	Lack of computing resources/Pulmonology	Deep learning	Present a novel, lightweight, and interpretable deep learning framework that discriminates COVID-19 infection from other cases of pneumonia and normal cases suitable for deployment in point-of-care and/or resource-constrained settings.
Jana et al., 2020 [36]	Patients	India	Lack of computing resources/Cardiology	Machine learning	Develop a smartphone-based portable continuous-wave Doppler ultrasound system for diagnosis of peripheral arterial diseases based on the hemodynamic features in a way that is more cost-effective and power-efficient, making it suitable for low-resource settings with limited energy and computing resources.
Hannan et al., 2023 [37]	N/A	United States	Emergency/Emergency medicine	Deep learning	Develop a deep learning-driven classifier that can aid medical professionals in diagnosing whether a patient has pneumothorax based on POCUS images. Design the classifier to perform in a mobile phone using little training data to train the model, making it suitable for low-resource settings such as emergency and acute-care settings.
Ekambaram and Hassan, 2023 [38]	Patients	South Africa	LMIC and rural/Emergency medicine	Bayesian machine learning	Propose a novel, Bayesian-inspired, iterative diagnostic framework that uses point-of-care-focused echocardiography to evaluate the conditions of patients with acute cardiorespiratory failure and suspected severe left-sided valvular lesions. This overcomes the current limitation that diagnostic protocols cannot perform sufficient quantitative assessments of the left-sided heart valves.
Khan et al., 2016 [39]	16 to 41-week-old fetuses	Norway	LMIC and rural/Obstetrics	Computer vision (OpenCV, Kalman-based tracker)	Develop an automatic method for localization of the presented section through the abdomen and measurement of the mean abdominal diameter (MAD) of a fetus designed to be operational in both traditional ultrasound settings and the rural areas of low- and middle-income countries.
Heuvel et al., 2019 [40]	Pregnant women	Ethiopia	LMIC/Obstetrics	Deep learning	Present a system that can automatically estimate the fetal head circumference (HC) from the point-of-care ultrasound image data obtained using the obstetric sweep protocol (OSP) to overcome the limitation of pregnant women in developing countries having no access to ultrasound imaging as it requires a trained sonographer to acquire and interpret the image.
Jafari et al., 2019 [41]	N/A	Canada	Lack of computing resources/Cardiology	Deep learning	Present a computationally efficient deep learning-based application for accurate left ventricular ejection fraction (LVEF) estimation. This application runs in real time on Android mobile devices that have either a wired or wireless connection to a cardiac POCUS device, making it suitable for a resource-limited environment.
Al-Zogbi et al., 2021 [42]	N/A	United States	Emergency/Pulmonology	Deep learning	Propose an autonomous robotic solution that enables point-of-care ultrasound scanning of COVID-19 patients’ lungs for diagnosis and staging through the development of an algorithm that can estimate the optimal position and orientation of an ultrasound probe on a patient’s body to image target points in lungs. This is useful in low-resource settings such as emergency situations where contact between healthcare workers and patients is not feasible (e.g., COVID-19 infection risk).
Blaivas et al., 2020 [43]	N/A	United States, Canada	Lack of computing resources/Various departments	Deep learning	Create and test a “do-it-yourself” (DIY) deep learning algorithm to classify ultrasound images to enhance the quality assurance workflow for POCUS programs to enable those in low-resource settings to leverage AI applications for medical images usually owned by large and well-funded companies.
Baloescu et al., 2020 [44]	N/A	United States	Lack of trained personnel/Emergency medicine	Deep learning	Develop and test a deep learning (DL) algorithm to quantify the assessment of B-lines in point-of-care lung ultrasound, which helps in diagnosing shortness of breath, a very common chief complaint in the emergency department (ED). This is useful in resource-limited settings where not enough experienced users are available as B-line identification and quantification can be a challenging skill for novice ultrasound users.
Cheema et al., 2021 [45]	Patients	United States	Lack of trained personnel/Cardiology	Deep learning	Present the novel use of a deep learning-derived technology trained on the skilled hand movements of cardiac sonographers that guides novice users to acquire high-quality bedside cardiac ultrasound images. This technology can have a role in resource-limited settings where cardiac sonographers are not readily available.
Blaivas et al., 2021 [46]	N/A	United States	Lack of data/Cardiology	Deep learning	Uses unrelated ultrasound window data (only apical 4-chamber views) to train a point-of-care ultrasound (POCUS) machine learning algorithm with fair mean absolute error (MAE) using data manipulation to simulate a different ultrasound examination. The outcome measured is the left ventricular ejection fraction. This may help future POCUS algorithm designs to overcome a paucity of POCUS databases.
Cho et al., 2024 [47]	Fetuses	South Korea	Lack of computing resources/Obstetrics	Deep learning	Proposes deep learning-based efficient automatic fetal biometry measurement method for the system-on-chip (SoC) solution. Results show feasibility in low-resource hardware settings such as portable ultrasound systems.
Zemi et al., 2024 [48]	N/A	United States	Remote/Oncology	Deep learning	Explores the feasibility of integrating artificial intelligence algorithms for breast cancer detection into a portable, point-of-care ultrasound device. Achieved a performance benchmark of at least 15 frames/second and suggests the usefulness of the proposed framework in remote settings.
Karlsson et al., 2023 [49]	N/A	United States	Lack of computing resources and LMIC/Obstetrics	Deep learning	Early detection of breast cancer is crucial for reducing morbidity and mortality, yet access to breast imaging is limited in low- and middle-income countries. This study explores the use of pocket-sized portable ultrasound devices (POCUS) combined with deep learning algorithms to classify breast lesions as a cost-effective solution. This study utilized a dataset of 1100 POCUS images, enhanced with synthetic images generated by CycleGAN, and achieved a high accuracy rate with a 95% confidence interval for AUC between 93.5% and 96.6%.
MacLean et al., 2021 [50]	COVID-19 patients	Canada	Lack of computing resources/Pulmonology	Deep learning	Introduces COVID-Net US, a deep convolutional neural network for COVID-19 screening using lung POCUS images. This network is a highly efficient and a high-performing deep neural network architecture that is small enough to be implemented on low-cost devices, allowing for limited additional resources needed when used with POCUS devices in low-resource environments.
Adedigba et al., 2021 [51]	COVID-19 patients	Nigeria	Lack of computing resources/Pulmonology	Deep learning	Develops a tele-operated robot to be deployed for diagnosing COVID-19 at the Nigerian National Hospital, Abuja, driven by a deep learning-based algorithm that automatically classifies lung ultrasound images for rapid, efficient, and accurate diagnosis of patients. The gantry-style positioning unit of the robot combined with the efficient deep learning algorithm is less costly to fabricate and is better suited for low-resource regions than robotic arms used in the status quo.
Pokaprakarn et al., 2022 [52]	Pregnant women	United States, Zambia	Lack of computing resources and LMIC/Obstetrics	Deep learning	Ultrasound is crucial for estimating gestational age but is limited in low-resource settings due to high costs and the need for trained sonographers. This study develops a deep learning algorithm based on the blind ultrasound sweeps acquired from 4695 pregnant women in North Carolina and Zambia, showing a mean absolute error (MAE) of 3.9 days compared with 4.7 days for standard biometry. The AI model’s accuracy is comparable to trained sonographers, even when using low-cost devices and untrained users in Zambia.
Karnes et al., 2021 [53]	COVID-19 patients	United States	Lack of computing resources and LMIC/Pulmonology	Deep learning	Introduces an innovative ultrasound imaging point-of-care (PoC) COVID-19 diagnostic system that employs few-shot learning (FSL) to create encoded disease state models. The system uses a novel vocabulary-based feature processing method to compress ultrasound images into discriminative descriptions, enhancing computational efficiency and diagnostic performance in PoC settings. The results suggest the ability of the FSL-based system in extending the accessibility of rapid LUS diagnostics to resource-limited clinics.
Viswanathan et al., 2024 [54]	Fetuses	United States, Zambia	Lack of computing resources and LMIC/Obstetrics	Deep learning	Develops a deep learning AI model to estimate gestational age (GA) from brief ultrasound videos (fly-to cineloops) with the aim of improving the quality and consistency of obstetric sonography in low-resource settings by the model, which outperformed expert sonographers in GA estimation and can flag grossly inaccurate measurements, providing a no-cost quality control tool that can be integrated into both low-cost and commercial ultrasound devices. This innovation is crucial for enhancing ultrasound access and accuracy, particularly for novice users in low-resource environments.
Zeng et al., 2024 [55]	COVID-19 patients	Canada	Lack of computing resources and lack of trained personnel/Pulmonology	Deep learning	Proposes COVID-Net L2C-ULTRA, a deep neural network framework designed to handle the heterogeneity of ultrasound probes by using extended linear-convex ultrasound augmentation learning. Experimental results show significant performance improvements in test accuracy, AUC, recall, and precision, making it an effective tool for enhancing COVID-19 assessment in resource-limited settings owing to its portability, safety, and cost-effectiveness.
Abhyankar et al., 2024 [56]	N/A	India	Lack of computing resources/Various departments	Machine learning	Presents an intelligent decision support system for point-of-care ultrasound imaging, emphasizing resource-limited healthcare settings. Utilizing a decision tree algorithm on a Raspberry Pi-powered portable ultrasound device enhances image quality and diagnostic accuracy by making informed decisions during image capture and processing. Continuous data collection and user input allow for adaptive learning and optimization, ensuring reliability and regulatory compliance. This system aims to provide cost-effective, high-quality ultrasound imaging, improving healthcare accessibility and quality in underserved areas.
Madhu et al., 2023 [57]	COVID-19 patients	India	Lack of equipment/Pulmonology	Deep learning	Proposes an optimized Xception convolutional neural network (XCovNet) for COVID-19 detection from POCUS images. Depth-wise spatial convolution layers are used to accelerate convolution computation in the XCovNet model, which performs better on POCUS imaging than on other models, including COVID-19 classification. The results of the trial demonstrate that the proposed technique achieves the best performance among recent deep learning studies on POCUS imaging. POCUS is a viable option for developing COVID-19 screening systems based on medical imaging in resource-constrained settings where traditional testing methods may be scarce and where CT or X-ray screening is unavailable.
